# Conflict and user involvement in drug misuse treatment decision-making: a qualitative study

**DOI:** 10.1186/1747-597X-3-21

**Published:** 2008-10-06

**Authors:** Jan Fischer, Joanne Neale, Michael Bloor, Nicholas Jenkins

**Affiliations:** 1 School of Environment and Development, The University of Manchester, Oxford Road, Manchester, M13 9PL, UK; 2 School of Health and Social Care, Oxford Brookes University, Jack Straws Lane, Marston, Oxford, OX3 0FL, UK; 3 Centre for Drug Misuse Research, University of Glasgow, 89 Dumbarton Road, Glasgow, G11 6PW, UK

## Abstract

**Background:**

This paper examines client/staff conflict and user involvement in drug misuse treatment decision-making.

**Methods:**

Seventy-nine in-depth interviews were conducted with new treatment clients in two residential and two community drug treatment agencies. Fifty-nine of these clients were interviewed again after twelve weeks. Twenty-seven interviews were also conducted with staff, who were the keyworkers for the interviewed clients.

**Results:**

Drug users did not expect, desire or prepare for conflict at treatment entry. They reported few actual conflicts within the treatment setting, but routinely discussed latent conflicts – that is, negative experiences and problematic aspects of current or previous treatment that could potentially escalate into overt disputes. Conflict resulted in a number of possible outcomes, including the premature termination of treatment; staff deciding on the appropriate outcome; the client appealing to the governance structure of the agency; brokered compromise; and staff skilfully eliciting client consent for staff decisions.

**Conclusion:**

Although the implementation of user involvement in drug treatment decision-making has the potential to trigger high levels of staff-client conflict, latent conflict is more common than overt conflict and not all conflict is negative. Drug users generally want to be co-operative at treatment entry and often adopt non-confrontational forms of covert resistance to decisions about which they disagree. Staff sometimes deploy user involvement as a strategy for managing conflict and soliciting client compliance to treatment protocols. Suggestions for minimising and avoiding harmful conflict in treatment settings are given.

## Background

In recent years, user involvement has become an important concept in health and social care policy and practice. Its origins and development have variously been related to the anti-psychiatry movement, the rise of consumerism, the emergence of self advocacy and pressure groups, the growth of community action, New Right policies, and the increase in public willingness to question expert knowledge in late modern society [1-5]. Today user involvement is commonplace in health fields as diverse as cancer treatment, mental health, learning disabilities and maternity services, with the research literature indicating that it improves service provision (e.g. [6]), empowers individuals (e.g. [7]) and is a democratic right and an ethical requirement (e.g. [8]).

Although user involvement has been relatively slow to develop within the UK drug treatment field, steady progress has been made and a strong user involvement movement now exists (as exemplified by Narcotics Anonymous, The Alliance, and Mainliners) [9]. Drug user representation at the level of national policy making remains poor, but local Drug (and Alcohol) Action Teams are involving users through user groups, user involvement co-ordinators, and user consultations. In addition, regional users' forums have been established, and the National Treatment Agency (NTA) in England has written user involvement clearly into recent policy documents, stating for example that 'Service users should be involved in all key aspects of decision-making in relation to their care' [10]. This paper focuses on the involvement of drug users in making decisions about their own treatment, a practice also referred to as client-centred treatment (e.g. [11-13]).

Implementing user involvement within the substance misuse field is, however, unlikely to be straightforward. Over the years, many authors have portrayed drug users as impatient, manipulative and aggressive, and identified hostility and anger as typical drug user characteristics [14-19]. Indeed, De Leon has argued that drug users 'often display an extreme sense of entitlement and exaggerated reactions to perceived unfairness, a need for immediate gratification in the form of instant answers, resistance through arguments, and a tendency to manipulate authority figures' [20]. Involving such individuals – particularly in treatment decision-making – seems prone to difficulty, especially if they breach treatment protocols and misuse treatment facilities [21]. Furthermore, a strong blame culture within the drug treatment field means that drug users are often seen as undeserving and not consulted despite policy statements [22].

In some treatment settings, particularly residential settings, staff may also actively provoke (and subsequently manage) conflict with drug users as part of the therapeutic process. Known as 'reality confrontation', the intention is to contain dysfunctional behaviours and enable clients to discover that they can tolerate uncomfortable emotional states and learn appropriate responses to difficult situations [23-26]. Such activity involves the repetitive depiction to clients of their behaviour as unacceptable, alongside the depiction of the community as a locale where other less pathogenic ways of behaving could be experimented with and adopted [27]. In addition, therapeutic communities for drug users are often (following the original 'Synanon' model [28]) hierarchically organised with a highly structured programme of activities and elaborate systems of rules. The expectation is that residents will fall foul of the rules and perform inadequately in the programme, but these failures offer valuable opportunities for therapeutic work [29].

Taking all the above factors into consideration, it seems reasonable to assume that involving drug users in making treatment decisions will create tensions, disagreements and even outright conflict between clients and staff. However, a broader reading of the sociological literature cautions against any simplistic assumptions. General writings on the doctor-patient relationship have long suggested that overt conflict in the healthcare setting is rare [30-32] and work on therapeutic communities for those with mental health problems indicates that dissenting clients will often use concealment and other forms of resistance in their interaction with staff, rather than resorting to open conflict [33]. As Goffman notes, residents of institutional settings will frequently avoid open conflict with staff by making secondary adjustments, such as working the system, taking back some small measure of control over their immediate environment and feigning to accept the negative views that staff have of them [34].

Certainly, user involvement has become a popular concept in substance misuse treatment policy and practice. Nonetheless, the existing literature does not provide a clear picture of whether and, if so, how involving drug users in treatment decision-making might result in conflict, how individuals might react to that conflict, and what the impact of any conflict might be. In this paper, we therefore seek to develop our understanding of user involvement by examining the extent, causes, responses to, and outcomes of conflicts occurring between the clients and staff of drug treatment services. We also consider whether conflict between clients and staff is always negative and how any negative forms might be prevented or reduced.

## Methods

The paper draws on data from in-depth semi-structured interviews conducted with 79 drug users and 27 treatment agency staff. The 79 drug users were recruited from two community drug treatment services (n = 19 and n = 20) and two residential rehabilitation agencies (n = 20 each). They included 53 men and 26 women, who were all interviewed within ten days of beginning a new treatment episode and who were all seeking treatment for their use of illicit drugs (predominantly opiates and/or stimulants). Their prior treatment histories were very varied (from non-existent to lengthy), with the clients of the residential services tending to report the most previous treatment episodes.

Contact details for all drug users were recorded at first interview and efforts were made to re-contact them after twelve weeks. In total, 59 were successfully re-interviewed (n = 14 and n = 15 from the two community services; n = 14 and n = 16 from the two residential services). Those who were not re-interviewed could not be traced at any known address, repeatedly failed to keep appointments with the interviewer or refused to participate in a second interview. Of the 29 community treatment clients who were re-interviewed, only 3 had left treatment at follow-up. In contrast, 16 of the 30 re-interviewed residential treatment clients had left treatment by their second interview. Most of these 16 individuals said that their treatment had ended for negative reasons, rather than because they had been ready to move on.

The 27 treatment agency staff who were interviewed were all nursing or care staff who had been selected to participate in the research because they were the designated key worker of one or more of the clients interviewed. Ethical approval for all interviews was granted by the Thames Valley Multi-centre Research Ethics Committee (approval number: 05/MRE12/48) and research was compliant with the Helsinki Declaration. All drug users and staff gave written informed consent prior to each interview and, during their first interview, all drug users consented to being re-contacted after twelve weeks for a follow-up interview.

Towards the end of each interview, drug users and staff were asked to engage with a developmental vignette, a research technique previously described in the drug misuse literature by Hughes [35]. The vignette told the story of a fictional drug user's treatment career and incorporated a number of scenarios with potential for conflict relating to treatment decision-making. These included the protagonist being offered a relatively limited methadone prescription, being forced to participate in group work, and being asked to own up to using drugs within a residential setting. Interviewees were asked to state how they thought the character would respond to each of these events. The vignette and more details on the research methods are reported elsewhere [9].

Both the semi-structured interviews and the vignette data were transcribed and coded with the aid of the software package MAXqda2. Codes from the interviews relating to past treatment experiences, experiences of the current treatment episode, and future expectations regarding the current treatment episode were exported into Microsoft Word files and systematically searched for evidence of conflicts relating to treatment decisions. These instances were then analysed thematically using Framework [36]. Responses to the vignette stages were similarly analysed and also used to appraise the reliability of the interview data. Although the client interviews were drawn from two time points, a longitudinal analysis is not presented here as we wanted to incorporate data from all of the clients' treatment experiences (previous and current). This was for two reasons: first, we wanted to maximise the amount of analysable data; second, we wanted to counter the likelihood that clients might give socially desirable responses when discussing their current treatment episode.

Preliminary findings indicated that 'conflict' was a very broad term that encompassed relatively trivial incompatibilities of opinion through to major disputes that resulted in treatment breakdown. After team discussions, we therefore decided to employ the terms 'actual conflict' and 'latent conflict' to further the analyses. We used the term 'actual conflict' to refer to those situations which had already resulted in open hostilities or clearly expressed differences of opinion. In contrast, we used 'latent conflict' to refer to situations that had not progressed into open disagreements but had the clear potential to develop into serious arguments *and *to describe signs of disagreement hidden below a veneer of consensus.

## Results

### The extent of conflict

During their first interview, most clients reported that they were not anticipating any conflicts in their current treatment episode. Furthermore, there was no indication that drug users from either the residential or community settings expected that staff would deliberately initiate conflict as part of the therapeutic/treatment process. Despite this, clients from the two residential units were more likely than clients from the two community services to report that future disputes might occur. These differences between service settings seemed to relate to two factors. Firstly, the residential clients – having generally had more previous treatments than the community clients – were more aware of the kinds of conflicts that could arise, particularly as they had often been referred to residential treatment after failing to stabilise in a community setting. Secondly, residential treatment is a more intense experience than community treatment and so more prone to generating stresses and strained interpersonal relations.

The follow-up interviews generally confirmed the clients' expectations, with fewer reports of conflicts in the community agencies than in the residential agencies. These findings were partially mirrored in the staff interviews. Although expectations of conflict varied substantially between individual staff members, residential workers appeared to expect a higher frequency of conflicts than those employed in community settings.

Given that most clients did not anticipate problems with staff at their first interviews, it was not surprising to find that they had not pre-prepared any strategies for dealing with disputes or disagreements. This is contrary to what one might have expected from the image of the impatient and manipulative drug user, with an extreme sense of entitlement, described in some of the literature discussed earlier. Instead, many treatment clients spoke of approaching their first treatment contact with openness and honesty, in the hope that all would go well and they would receive much-needed help and support. For example, the following statement was made by a female community client who had accepted her mother's advice regarding the importance of being honest with services:

One thing my mum said is just to make sure I'm honest, do you know what I mean? There's no point in me coming down to somebody unless I'm being honest, 'cos otherwise I'll not get the help I need, do you know what I mean? So there's no point in me trying to hide anything. [community client, first interview]

Additionally, when clients were asked what they expected their first meeting with the treatment agency staff would be like, most said that they had never thought about the issue. As this male community agency client explained:

I have never really thought about it. I just take it as it comes. I shall be as polite as I can and talk as normal as I can to them and hope they respect me. [community client, first interview]

Those who had given the matter of the first treatment encounter any consideration tended to report that they wanted, and were expecting, to be guided by staff rather than to take the lead themselves. This was because they perceived staff as being the experts who would know the best course of action to take and be able to allay their fears and anxieties. Moreover, staff who were themselves ex-drug users were deemed to have the greatest knowledge, understanding and credibility. Similar findings emerged from the vignette. When interviewees were asked whether the therapist in the fictional story was right or wrong to insist that the protagonist attend a group session against his will, most clients thought that attendance should be compulsory. Frequently, they justified this by reference to the therapists' expertise or a belief that group work must be beneficial if it was a formal part of the treatment programme:

I was apprehensive at the very beginning *[about group sessions] *but once you do start attending them you do realise where the therapists are coming from. Most therapists are actually ex-users and they know what they are talking about. So, I believe that they are right. [residential client, first interview]

Although the employment of ex-users varied substantially between our four treatment agencies (with one residential agency employing almost exclusively ex-users and one community agency employing none), staff interviews often confirmed that ex-users as staff enjoyed greater credibility than staff who were not ex-users. Furthermore, despite a general acceptance of user involvement, staff seemed to agree with clients in favouring a staff-led service:

They *[clients] *certainly have a say in it in the sense that within a week you have to agree a detailed treatment plan with the patient and with the head of treatment here. If the patient thinks it is wrong, they can certainly say so. The patient can't really dictate more than that. I mean, they can't come in here with their own ideas about what they can and can't do, but they do certainly have a say. [residential staff member]

This symmetry of client and staff viewpoints was less evident at the second client interviews. Although the majority of clients still believed that clients should defer to staff expertise, a minority now felt that more user participation in treatment decisions would be beneficial:

I think it should all be decided by them *[staff]*. Because, at the end of the day, at the start of treatment you're not really capable of deciding what is best for yourself. [residential client, first interview]

I just think, if the people *[clients] *had more say, they'd feel more comfortable in the things, especially for the newer people. [same client, follow-up interview]

About half of those who were re-interviewed reported that they had experienced conflict (actual or latent) within their ongoing, or by then terminated, treatment. Some clients referred to isolated, low-level latent disputes, but a few stated that they had disagreed with staff in a more or less open way on a number of occasions. The majority of clients who had left treatment between their first and second interview had experienced conflict of some kind and this conflict had often related to their treatment ending. Amongst those clients who remained in treatment at their second interview, initial enthusiasm for open communication had sometimes diminished because of a conflict experience (the causes of which are examined below).

### The causes of conflict

In the early stages of treatment, many of the causes of conflict discussed by clients related to aspects of the treatment programme that angered or irritated them because they did not understand them or find them helpful. In residential rehabilitation settings, clients commonly complained about 'silly' or 'petty' rules and particular programme procedures. These included restrictions on music, television and phone calls; not being allowed to consume food brought onto the premises; and not being allowed to go to one's room alone. Examples of procedures that irritated clients were unnecessary domestic chores; being constantly chaperoned; and having to participate in group work. This interviewee, for example, describes how some of these rules had made her very angry:

I didn't know I wasn't allowed to bring anything up, a hi-fi, whatever...And I went out and spent money and bought cranberry juice, things that I drink. *[Staff member] *took half my things off me. I was angry about that...And I was feeling really lonely, really hurt. I just wanted to go to my room and cry. And then I was told you have got to get permission to do that. [residential client, first interview]

When clients felt that they did not understand the reasoning behind rules and procedures, low level discontent and irritation (latent conflict) sometimes escalated into actual conflict. Conversely, clients' annoyance at rules and procedures very often subsided or disappeared if the reasons for such arrangements were clearly explained to them. Thus, in the residential services, frustration at not being allowed to go to one's own room could, with explanation, be reconstructed as positive encouragement to participate in the community. Equally, pointless cleaning duties could be reinterpreted as keeping physically and mentally active. This male client described how he had initially thought that not allowing new residents to listen to music was a 'stupid rule'. Nonetheless, he had come to view it as a 'good idea' after someone had clarified to him the reasoning behind it:

I started listening to dance music and I would think of all the good times I had had with dance music on valium and smoking a joint. So for the first six weeks there's no music or television. It's just really focusing on you, because these things make you isolated if you sit and listen to music, and really you should be in the community and talking to people and focusing on you, you know what I mean? So it is a good idea. [residential client, first interview]

Even though rules, and particularly the highly structured nature of residential treatment, remained a source of discontent for clients at follow-up, those who remained in treatment mostly came to accept that there was no point in arguing about the content of treatment rules as these would never be changed. Instead, client unhappiness seemed to shift to the way that rules were enforced. Clients – particularly residential clients who had themselves been discharged from treatment by staff because of a rule violation – maintained that some staff were too strict or too inflexible in their enforcement of rules. Meanwhile, some community service clients complained that appointment systems were overly strict or that they had been given stricter treatment regimes than others (for example, they had had to consume their medication under supervision or had been subject to frequent urine testing). The strongest resentments, however, seemed to arise when staff imposed different penalties on different clients for the same or similar rule violations:

Just the way the place is run, but it is annoying and it just causes resentment. It is like when people go off project drinking. Oh, you go off project drinking, come back, you either come back razzled or you have got this problem going on: 'Okay, we will slap you and don't do it again.' Somebody else does it and it is like: 'Sanction. Seven days notice to quit.' Do you know what I mean? ... I personally *[think] *it should be one rule for all. [residential client, follow-up]

Another common source of latent, and some actual, conflict reported by clients was negative staff attitudes and negative staff behaviour. Negative attitudes included staff being uninterested, unsympathetic or looking down on clients. Negative behaviours included staff being remote and uninvolved, not listening to clients, and failing to act – particularly failing to do things that they had promised the client they would do. At the follow-up interviews, only a handful of clients reported that staff had deliberately provoked confrontation and most did not see this as a positive therapeutic measure. On the contrary, staff negativity was overwhelmingly perceived by clients as unhelpful and unconstructive. Indeed, it was occasionally cited as a reason why they had dropped out of treatment.

For a very small number of drug users, actual conflict had occurred when they had wanted a particular treatment but been refused it. This might have happened if a client had requested a treatment which the agency did not provide, or for which the agency had considered them unsuitable, or if the desired treatment had been particularly expensive or difficult to obtain (such as a residential treatment with limited places). For example, this female client had for a time been refused a referral into residential treatment because she was not considered sufficiently stable:

It really sent me off my head, because I was like 'how can you not be stable enough for rehab?' And that was her words to me. But she didn't prescribe anything, do you know what I mean? And I was like 'I'm crying out for help here, why are you not helping me?' [residential client, first interview]

More commonly, latent conflict was evident when drug users anticipated that they would be given a treatment that they particularly disliked, especially methadone. Overt conflict was usually avoided in these situations because the disliked treatment was never actually offered, the client had simply declined it when offered, or the client had conceded and accepted it without complaint. Latent conflict was similarly apparent in the vignette when respondents were asked how the protagonist would feel about being offered only a relatively limited methadone dosage. Many clients reported that this would likely be a source of tension, but often qualified their responses by acknowledging that other drug users would not necessarily be unhappy (or that they would see the offer as a starting dose rather than all they could expect to receive).

Those in residential services additionally stated that the first few days of a residential programme could be particularly prone to arguments, disputes and conflict more generally. This was because clients were often stressed and irritable if they were feeling disorientated, vulnerable, and lonely. Moreover, this was likely to be exacerbated if individuals were undergoing a period of rapid detoxification, since the withdrawal process frayed tempers and made individuals behave unreasonably. Indeed, there was a general feeling that residential clients could not be expected – by staff or others – to be reasonable at such a testing time. Reflecting this, some clients reported that the demands of their detoxification had contributed to them dropping out of treatment prematurely and some called for more or better medication to address the severity of their withdrawal symptoms:

I think there should be medication. They knew that, I think, but four weeks I was up like a baby; stayin' up every night, every night mostly. That's why I got sick o' it. If I'd maybe got a couple o' nights sleep I might have stayed. [residential client, follow-up interview after leaving treatment]

Less frequently, drug users acknowledged that their own bad behaviour and/or negative states of mind were causes of conflict in treatment. Some interviewees thought that their own 'attitude problems' and personal inclinations to 'argue back' at authority were likely to be the source of conflict in their current treatment. Others reported that they had previously dropped out of treatment because they had not really been 'ready' to address their problem. As this client remarked:

It's been my fault, you know...Basically because it comes down to me, you know. I've either relapsed or something...I've had to leave the projects, so usually been down to myself. [community client, first interview]

Staff also identified lack of compliance, rule violations and inappropriate client behaviour as causes of conflict. Indeed, some staff members felt that clients were not truthful about many aspects of their lives and some stated that they would challenge clients who gave contradictory accounts of their treatment progress or who appeared to tell untruths. Despite this, the most common causes of conflict identified by staff were clients' unrealistic expectations about treatment. These included clients expecting medications that were not on offer to them, or that were only offered at low dosage, and not appreciating how long it would take between first seeking treatment and achieving relative stability:

One of the typical sources of conflict is about how soon they think things happen. [community staff member]

Finally, some staff and clients pointed out that conflict inevitably occurs in treatment settings because it is human nature to disagree and argue, especially in residential units where large numbers of people live together. In these cases, our respondents did not distinguish between conflicts that occurred between staff and clients, conflicts that occurred between clients and other clients, and even conflicts within the staff team. Such statements provide a useful reminder that it can never be possible to prevent all conflict in the treatment setting.

### Drug users' strategies for dealing with conflict

As indicated previously, most clients had not prepared a strategy for dealing with any conflict occurring in their current treatment episode. However, when asked what they would do if a serious disagreement arose, most reported that they would try to resolve it by communicating with staff. In this regard, they usually stated that they would explain their position or their side of the story so that staff would better understand things from their point of view. Others stated that they would apologise if they had behaved badly or attempt to talk their way out of a difficult situation. Significantly, many emphasised that they would try to reach a compromise with staff, a finding which again runs counter to the notion of the uncooperative and demanding client. When asked how he would resolve any conflict that might occur in his current treatment, this client responded:

Talking. I talk, me. I resolve my problems by talking. [community client, first interview]

Other clients maintained that they would use avoidance tactics as a means of dealing with potential sources of conflict. This might be avoiding a particular staff member or another service user, refusing a particular element of a treatment programme (such as methadone), or even leaving treatment altogether. This residential client described how he planned to avoid trouble in his current treatment episode by keeping a low profile:

I'm having my ups and downs myself at the moment. But I think, as I say, the people seem alright. So I'll just keep my head down and get on with my own thing and I should be alright. [residential client, first interview]

Staff equally identified negotiation – albeit usually within a framework of rules – as a preferred method for resolving conflicts. Thus, staff described how formal rules and procedures could be used to discipline noncompliant clients and so direct the client's behaviour. Whilst both residential and community staff members felt that some rules were not open to negotiation, the residential settings had the most formalised and elaborate structures. These included written rules about acceptable and unacceptable behaviour and established procedures for taking interpersonal disputes to the wider house community for discussion. Clients of the two residential agencies also reported that they would actively use the formalised structures established in those services to address any actual or potential conflict. Indeed, even clients who had only been in the house for one or two days often had a very good grasp of how the house system could be deployed in this way. As one residential client commented when asked how he might deal with any future conflict:

Use the system. Use the tools and diplomatically. [residential client, first interview]

In contrast, community clients were often less aware of – or less willing to use – formal procedures for addressing grievances, such as the complaints procedures offered by the National Health Service. During follow-up interviews, a number of residential clients identified group work as a useful mechanism for dealing with conflicts in a constructive way. Others felt that group work was at times intimidating and frustrating, and some noted that acceptance of group work was a gradual process as fears were substituted by appreciation of peer advice:

The therapeutic groups are good. You've got people in your group to help you. They see you as you are and it's like they're telling you what's wrong. Me myself was kinda putting barriers up: 'That's not me!' and 'Don't you dare say that about me!' you know? But, after a wee while you kinda sit and you look at it, what they see, because they're actually seeing ya. [residential client, follow-up interview]

Responses to the vignette, meanwhile, indicated that when disputes occurred between staff and clients, many clients believed that they had little option but to concede to staff if they wanted to stay in treatment. In addition, it appeared that honesty could play an important role in dealing with potential conflict. For example, the majority of clients thought that the fictional treatment character in the vignette would be more likely to own up to, than to lie about, using heroin whilst in residential rehabilitation. This was because they felt that the main factors motivating him would be to stay in treatment, do what was morally right, and not to let his house mates down. Showing honesty – rather than arguing or lying – was considered the best ways of proving commitment to the programme, learning from mistakes, and ultimately securing a better treatment outcome. In stark contrast, most staff members expected that the fictional drug user would keep his rule violations secret because he would be afraid of loosing his place in the programme.

Despite their first interview statements about how they would respond to conflict and disagreements by communicating and negotiating, the follow-up interviews showed that users had often not succeeded in addressing problems in a calm, open and diplomatic way. Instead, clients often simply kept quiet about their problems or side-stepped conflict by making 'secondary adjustments', such as purchasing drugs illicitly from the street if they could not obtain them on prescription. Thus, latent conflict never developed into open dispute and client acquiescence could mask hidden dissent:

I mean she is the doctor at the end of the day and if she don't think it right to give them [Valium] to me....So maybe she thinks that I...but if she doesn't give them to me and I go and buy them off the streets anyway so... [community client, second interview]

Other conflicts – albeit rarely – resulted in shouting matches and verbal abuse. An example is provided by this client, who later regretted his response to an argument in which he had been told to sit on a bench (a technique used within the residential agency to calm clients and address disobedience):

I had something put to me. I didn't agree with it and I got told to take the bench and I refused to take the bench and told them to stick it where the sun doesn't shine, basically.... It was out of order; it was very wrong doing that. [residential client, follow-up interview]

### Conflict outcomes

When clients reported on conflicts arising in both their current treatment episode and any episodes of treatment they had had in the past, a number of outcomes seemed possible. One of the most common was the termination of treatment, arising either because the client had left treatment of his or her own accord or because staff had decided to cease providing treatment to them. The kinds of conflict that resulted in clients leaving treatment of their own accord were likely to be latent, relating to such factors as clients disliking the service or aspects of it, feeling that staff had a negative attitude towards them, or perceiving that the treatment was not helping them sufficiently. The kinds of conflict that resulted in staff deciding to end treatment tended to be actual conflicts, resulting from the client being caught tampering with urine samples, using drugs, or selling drugs. For example, this client recalled how he had been discharged from a residential service for taking drugs brought in by another client:

I basically said to them that the whole reason I was there was because I couldn't just say no. I couldn't resist temptation and if a drug was in front of me, I was going to use and that was the whole reason I got asked to leave. [residential client, follow-up interview after discharge]

A further outcome was that staff simply decided on a response, position or course of action and refused to compromise with the client. Thereafter, the issue was not discussed again. In such cases, the staff member would tend to rely on agency rules or policy to uphold their position and the client would defer to them because of their desire for treatment or because they appreciated that they could not win an argument with a professional. In practice, however, the conflict was never actually resolved and so it effectively remained a latent dispute:

Well, at the end of the day, they are professional. They always have the last say. There was nothing that kinda blew up, nothing major, just a couple of disagreements. [residential client, first interview]

For residential clients in particular, conflict could be addressed by appealing to the governance structure of the residential agency. This meant that conflicts, rule violations or other issues that might disrupt treatment were submitted to a group for discussion or that the agency had a rule to deal with particular transgressions, although staff often retained the role of the final decision-maker in both instances. A number of clients seemed to value the process of tackling concerns in a group environment, and some found it therapeutic to address their disputatiousness with others – that is, to be subject to the 'reality confrontation' described earlier:

[Recently] I've been quite angry and 'don't you tell me what to do'...I am 27 and I've got to stop acting so young....I do need my arse kicked basically, I do. [residential client, second interview]

However, others perceived therapeutic groups as intimidating, as this client explained:

Basically it's jist....well what happens in them *[groups] *is people get put up for concern like whether it be health or if they're doin' somethin' wrong so that it's voiced in front of like the whole community, so that we're aware and we can help that person. Athough sometimes it disnae feel like that. Ye think they've got the firing guns out at ye. [residential client, follow-up interview]

Another less frequent outcome of conflict was that clients managed to broker a compromise with staff. In these situations, clients generally reported being very happy with the settlement reached and seemed to feel that it had genuinely resolved the conflict. Several staff members also indicated that they preferred a negotiated outcome over any other. In addition, a very small number of clients reported that they had managed to resolve a conflict entirely in their own favour. This had, however, only occurred in situations where the underlying problem was a misunderstanding relating to a technical issue (such as an incorrect prescription) rather than a substantive difference of opinion.

The very limited degree to which clients were able to resolve disputes in their favour is mirrored by staff who reported that skilful staff work involved eliciting client consent for staff decisions:

It's that informed choice, um, having to have enough knowledge of what they're actually wanting to talk about and be able to show them the pros and cons of each....and then make them think again about what will work for them. And say: 'Well, you've told me you've done this in the past and that in the past and it hasn't worked and I'm offering this and that and the other....so you need to make that choice. But I would think that the best way from what you're telling me is to go this way, rather than that way. [community staff member]

For some staff members, it was evident that conflicts could serve as a very useful occasion for therapeutic work. This was because they provided the staff member with opportunities for showing the client Cooley's 'looking glass self' [37]. That is, the staff member could reflect back to the client the image that staff and other clients had of them and their behaviour. Equally, conflicts allowed staff the chance to convert the client to – and get them to agree with – the staff's view of the client's difficulties and needs.

## Conclusion

Our study is not without limitations. For example, we did not collect any ethnographic or observational data. This limits our ability to adjudicate between discrepant client and staff accounts of conflict. In addition, our second interviews took place only three months after treatment had started. In some residential agencies, a client is barely seen as settled-in after this time period and some confrontational aspects of therapy may only commence at a later stage. Also, we were not able to re-interview all of the clients. Those not re-interviewed were often no longer in contact with our treatment services and so it is possible that they were discontented service users who might have reported more conflicts than those who were re-interviewed. Despite these weaknesses, our analyses still offer new insights into conflict and user involvement and have potentially important implications for policy and practice.

As we discuss at the start of our paper, there has been an increased acceptance of user involvement within the addictions in recent years. However, involving drug users in making decisions about treatment has the potential to generate conflicts which could ultimately undermine the treatment process. Clients are often viewed as dishonest and manipulative, staff may sometimes actively provoke conflict with drug users as part of a broader therapeutic process, and some treatment agencies are hierarchically organised and rule-bound in ways that inhibit flexibility to individual needs. Beyond this, aspects of service provision are dictated by policies and funding mechanisms that transcend any particular service, and some treatment decisions (for example, dose levels in substitute prescribing) might be viewed as inherently contentious.

Perhaps unsurprisingly then, our study found evidence of conflict in both residential and community drug treatment settings, particularly during the initial detoxification stage of a residential programme when clients were withdrawing and feeling vulnerable, confused, stressed and isolated. The types of conflict identified were diverse, and ranged from relatively trivial differences of opinion through to more serious disagreements that resulted in clients leaving, or being told to leave, treatment prematurely. Although overt conflict involving shouting and verbal abuse was relatively infrequent, forms of 'latent' conflict – that is, negative experiences and problematic aspects of treatment that could potentially escalate into overt disputes – were common.

Notwithstanding the above, our study found little evidence that drug users were manipulative, overdemanding, aggressive or impatient. On the contrary, they seemed positive and co-operative at treatment entry and were often hoping and expecting to be guided by staff in treatment decision-making. Indeed, many wanted to be honest and open with the professionals who were helping them and appeared keen to discuss and resolve any problems that might arise. Beyond this, some clients believed that they had to accept staff decisions if they wanted to receive support and others adopted non-confrontational forms of covert resistance (rather than open discontent) when faced with treatment decisions about which they were unhappy.

Such findings are broadly consistent with sociological literature which has suggested that overt conflict between clients and healthcare professionals is rare [30-32] and clients will often behave in ways that demonstrate resistance to staff power, but without any actual open demonstration of dissent [33,34]. Furthermore, our data showed how staff were on occasions able to avoid conflict in treatment encounters by skilfully deploying user involvement as a strategy for soliciting client compliance. For example, staff sometimes offered reasoned grounds for treatment decisions in order to increase the likelihood of client adherence to treatment protocols; provided structured opportunities to discuss decisions on the governance of treatment facilities in order to increase client acceptance of the social structures of treatment; and provoked dispute through elaborate hierarchies and house rules in order to provide occasions for reality confrontation.

From this, we can conclude that involving drug users in decisions about their treatment – by, for example, listening to them, consulting with them, negotiating with them, questioning them, challenging them and even provoking them – does not automatically result in conflict. Moreover, when conflict (actual or latent) does occur, this will not necessarily be negative or detrimental to treatment processes or outcomes. However, we must also accept that conflict can be unproductive, may damage treatment relations and, in the worst case scenario, can lead to treatment breakdown. Accordingly, it is important to consider how user involvement might be implemented so that harmful forms of conflict can be minimised and avoided whenever possible.

In our research, negative conflict (latent and overt) often occurred because clients did not understand aspects of the treatment programme, particularly the reasoning behind rules and procedures. Equally, they became resentful when staff imposed different penalties on different clients for the same or similar rule violations. Such findings suggest that detrimental forms of conflict might often be avoided if staff spent more time communicating and explaining treatment issues to their clients. Supporting this, many drug users expressed a clear desire to talk to, and negotiate with, staff. However, in practice, they often failed to voice their concerns calmly and reasonably. Arguably, therefore, user involvement might also be improved if services could offer clients more basic training in communication skills, stress management and conflict resolution.

Drug users' criticisms of particular treatments (such as methadone), meanwhile, revealed that some individuals would rather abandon treatment altogether than accept a service to which they were categorically opposed. This implies that services are more likely to be successful if clients have some choice about, and involvement in deciding, what treatment/s they actually receive. Despite this, we live in a world of finite resources and there may be very good reasons why an individual cannot receive a particular form of support. Again, in such situations, negative conflict might be minimised or avoided if clients were given clearer explanations regarding why a particular treatment option could not be received and/or if staff focused more on trying to manage and moderate any unrealistic expectations which clients appeared to hold.

Interestingly, some clients acknowledged that their own bad behaviour and negative states of mind, including not really being 'ready' to address their drug problems, had caused conflicts with staff. Coping with argumentative drug users and ascertaining whether or not someone is genuinely psychologically and emotionally 'ready' for a particular treatment will inevitably be difficult and call for a high level of staff sensitivity, skill and understanding. Staff must also recognise that drug users are not inevitably disruptive and that those who are being difficult may simply be reacting to their own stressful life circumstances. This seems particularly important in residential services, especially when clients are undertaking detoxification.

Our findings have shown that staff do manage conflict situations, including sometimes successfully converting the client to the staff's point of view. Yet, it has also been evident that many conflict situations are not successfully managed, causing clients to feel angry and resentful, but also contributing to them breaching treatment protocols and leaving treatment prematurely. Since many drug users highlighted staff negativity towards them as a source of actual and latent conflict, those working in drug services might require more training to enable them better to understand, and be more sympathetic towards, the circumstances, motivations, and stresses that new treatment entrants commonly face. Arguably, if drug users felt more respected by service staff – particularly in the early stages of treatment – they might be more willing to engage positively with treatment protocols and feel and behave more like genuine partners in the treatment process.

## Competing interests

The authors declare that they have no competing interests.

## Authors' contributions

JF assisted in the late design stages of the study, collected and analysed data, and drafted the manuscript. MB and JN designed the study, analysed data, and participated in drafting and revising the manuscript. NJ collected and analysed data, and commented on the draft. All authors read and approved the final manuscript.
